# PUBLISHER’S NOTE

**DOI:** 10.1080/20008198.2019.1577397

**Published:** 2019-02-12

The following article was originally published in issue 9(S3) (Special Issue: Complementary and integrative interventions for PTSD). It has now been moved to standard issue 10(1):

Fear extinction learning improvement in PTSD after EMDR therapy: an fMRI study

Pierre-François Rousseau^a^, Myriam El Khoury-Malhame^b^, Emmanuelle Reynaud^c^, Sarah Boukezzi^c^, Aïda Cancel^c^, Xavier Zendjidjian^d^, Valérie Guyon^d^, Jean-Claude Samuelian^d^, Eric Guedj^e^, Thierry Chaminade^c^ and Stephanie Khalfa^a^

^a^Laboratoire de Neurosciences Sensorielles et Cognitives, UMR 7260 CNRS, Marseille, France; ^b^School of Arts and Sciences, Neurosciences, Neuropsychology, Lebanese American University, Beirut, Lebanon; ^c^Timone Institute of Neuroscience, UMR 7289 CNRS, Marseille, France; ^d^Department of Psychiatry, La Conception University Hospital, Marseille, France; ^e^Biophysics and Nuclear Medicine Department, Timone Hospital, Marseille, France

DOI: 10.1080/20008198.2019.1568132

